# A Stakeholder-Based Analysis of Factors Influencing the Development of Grid-Forming Microgrids: A Partial Least Squares SEM Approach

**DOI:** 10.3390/bs16050641

**Published:** 2026-04-24

**Authors:** Chao Tang, Jiabo Gou, Xiaoqiao Liao, Jinhua Wu, Hongning Chu, Qingming Wang, Jiaming Fang, Shen Yan

**Affiliations:** 1 State Grid Panzhihua Electric Power Supply Company, Panzhihua 617067, China; tangc121x@sc.sgcc.com.cn (C.T.); goujb6734@sc.sgcc.com.cn (J.G.); liaoxq4857@sc.sgcc.com.cn (X.L.); wujh7216@sc.sgcc.com.cn (J.W.); chuhn0056@sc.sgcc.com.cn (H.C.); wangqm6414@sc.sgcc.com.cn (Q.W.); 2 School of Economics and Management, University of Electronic Science and Technology of China, Chengdu 611731, China; jmfang@uestc.edu.cn

**Keywords:** grid-forming microgrids, stakeholder theory, influencing factors, PLS-SEM

## Abstract

The deployment of grid-forming microgrids has attracted growing attention as a pathway toward improving energy system resilience and supporting low-carbon transitions in decentralized power systems. However, the relative influence of distinct stakeholder groups on microgrid development performance remains inadequately understood in the extant literature. Grounded in stakeholder theory and informed by behavioral economics, this study develops and empirically tests a stakeholder-based framework that examines the effects of government support, investor participation, user acceptance, and utility participation on microgrid development performance. Survey data were collected from 200 stakeholders engaged in microgrid-related activities and analyzed using consistent Partial Least Squares Structural Equation Modeling (PLS-SEM). The structural model accounts for a substantial proportion of the variance in microgrid development performance (R^2^ = 0.647). The quantitative results indicate that all four stakeholder constructs exert statistically significant positive effects on microgrid development performance. Investor participation emerges as the strongest driver (β = 0.399, *p* < 0.001), followed by user acceptance (β = 0.190, *p* < 0.001), government support (β = 0.175, *p* = 0.015), and utility participation (β = 0.170, *p* = 0.003). Interpreted through a behavioral economics lens, these findings demonstrate that development performance is governed primarily by behavioral and perceptual factors, namely capital confidence, risk tolerance, and demand-side acceptance, rather than by technical preparedness alone. Conventional assumptions of linear adoption driven by technical superiority are therefore insufficient to account for observed development outcomes in complex, decentralized energy systems. This study advances a stakeholder-centered and behaviorally grounded understanding of grid-forming microgrid development and offers empirical guidance for designing governance frameworks that align regulatory structures with market and user behavioral dynamics.

## 1. Introduction

The global energy system is undergoing a pronounced structural transformation, driven by climate change mitigation commitments, growing concerns regarding energy security, and the rapid expansion of renewable energy technologies. According to the International Energy Agency’s Electricity Mid-Year Update 2025, wind and solar are currently the fastest-growing components of global electricity supply. Their combined share of total generation is projected to increase markedly in the near term, with renewables collectively are expected to surpass coal as the leading generation source by 2025 or 2026. By 2027, the International Energy Agency (2025) projects that approximately 39.14% of global electricity will be generated from renewable sources. This trajectory reflects a fundamental reorientation of the global energy mix toward low-emission technologies and reinforces the strategic importance of distributed generation and grid-forming microgrids in improving grid flexibility, reliability, and local energy autonomy. Concurrently, the structural limitations of centralized power systems have become increasingly apparent, as extreme weather events and escalating electricity demand have exposed vulnerabilities that conventional grid architectures are ill-equipped to address. These pressures have prompted policymakers and practitioners to pursue decentralized and adaptive energy solutions capable of maintaining stable operations under uncertain and variable conditions (D. Brown et al., 2020).

Grid-forming microgrids have emerged as a particularly promising class of decentralized energy systems among the available alternatives. Unlike conventional grid-following topologies, grid-forming microgrids can operate autonomously from the main grid while actively regulating voltage and frequency. Through the coordinated integration of renewable generation, energy storage, advanced power electronics, and flexible demand management, these systems support higher levels of renewable penetration and greater operational resilience (Hatziargyriou, 2014). A subset of these configurations, commonly referred to as “water–solar–storage–direct–flexible” architectures, combines hydropower, solar photovoltaics, storage systems, direct current distribution, and adjustable loads, offering measurable reductions in energy conversion losses alongside improvements in local energy balancing. Despite these documented technical capabilities, the deployment of grid-forming microgrids in practice remains geographically uneven and operationally constrained, indicating that technical readiness alone does not determine development outcomes.

This observation is consistent with broader theoretical perspectives on energy transitions. Recent scholarship characterizes such transitions as inherently socio-technical processes, shaped not only by engineering feasibility but also by institutional arrangements, market incentives, and human behavior (Geels, 2019). Empirical evidence supports this view: inadequate investment confidence, limited user acceptance, fragmented governance structures, and misaligned utility incentives are identified as recurring contributors to delays and underperformance in renewable energy projects (Polzin et al., 2019; Sovacool & Ratan, 2012). Accordingly, stakeholder involvement has emerged as a critical determinant of energy system outcomes. Governments, investors, users, and utilities each bring distinct objectives, resources, and risk perceptions that collectively shape the trajectory of energy infrastructure projects.

Despite the relevance of these considerations, the majority of extant microgrid research has focused on cost–benefit evaluations, control system design, and technical optimization. The behavioral and stakeholder-driven factors that govern microgrid development performance have received comparatively limited scholarly attention. Prior research has examined specific dimensions, such as public acceptance of renewable energy technologies or the effect of policy instruments on investment decisions, but typically in isolation rather than within an integrative analytical framework. This limitation is particularly consequential for grid-forming microgrids, whose operational complexity demands a high degree of coordination across investment, regulation, user engagement, and utility integration. Accordingly, a comprehensive analytical framework that simultaneously examines these dimensions is needed to advance understanding of what drives development performance in these systems. While existing research has provided valuable insights into individual dimensions such as policy effects on renewable energy investment or public acceptance of clean technologies, these studies remain largely fragmented and descriptive in nature. As a result, they offer limited explanatory power in capturing how multiple stakeholder groups jointly and differentially shape microgrid development outcomes. This limitation constrains the ability to move beyond isolated factor analysis toward an integrated and quantitatively grounded understanding of stakeholder interactions in grid-forming microgrids.

The present study responds to this need by developing and empirically testing a stakeholder-based framework that identifies and quantifies the determinants of grid-forming microgrid development performance. Drawing on stakeholder theory and the socio-technical transitions literature, the study conceptualizes government support, investor participation, user acceptance, and utility participation as four distinct explanatory constructs shaping development outcomes. Survey data collected from 200 microgrid-related stakeholders are analyzed using PLS-SEM to assess the relative influence of each construct and to examine how institutional and behavioral interactions collectively shape system-level performance. By shifting the analytical orientation from purely technical considerations toward stakeholder-driven mechanisms, this study contributes a more comprehensive understanding of decentralized energy transitions and offers empirical guidance for governance design and policy formulation in the microgrid sector.

## 2. Literature Review

### 2.1. Grid-Forming Microgrids and Their Development Challenges

Driven by the dual imperatives of global climate change mitigation and energy security, energy systems are accelerating their transition toward low-carbon and decentralized structures (Ajaz & Bernell, 2021). The large-scale integration of distributed energy resources, exemplified by wind and solar power, poses significant challenges to the stability and flexibility of existing power grids. Against this backdrop, microgrids—capable of integrating distributed generation, energy storage, and loads to achieve self-control, protection, and management—are regarded as a key solution for enhancing distribution system resilience and promoting renewable energy integration (Hamidieh & Ghassemi, 2022; Kostenko & Zaporozhets, 2023).

Microgrids can be categorized into various types based on current type, voltage level, and operating mode (Khan et al., 2024; Mousa et al., 2023; Ortiz et al., 2020). By current type, they include AC microgrids, DC microgrids, and hybrid AC/DC microgrids; by voltage level, they are classified as low-voltage and medium-voltage; and by operating mode, they are divided into grid-connected and islanded microgrids. Among these, grid-forming microgrids specifically refer to systems possessing grid-forming capabilities. Their core lies in employing “grid-forming” inverters capable of autonomously establishing and regulating grid voltage and frequency, mimicking the operational characteristics of synchronous generators (Anttila et al., 2022). Compared to traditional “grid-following” microgrids, grid-forming microgrids can independently establish a stable power “backbone” under extremely weak grid conditions or even in islanded operation without a main grid. They provide essential voltage and frequency support, significantly enhancing system stability and reliability during weak connection or islanded operation (Anttila et al., 2022; Pogaku et al., 2007). Grid-forming technology empowers microgrids to actively support the grid rather than passively follow it. It enables inertia response, damping oscillations, and rapid frequency/voltage regulation, effectively addressing system strength degradation caused by high proportions of inverter-connected sources (Lasseter et al., 2019; Pattabiraman et al., 2018). Therefore, grid-forming microgrids represent a more technologically advanced and functionally autonomous “next-generation” microgrid form, serving as one of the foundational technologies for constructing future highly resilient, intelligent distribution networks (Hossain et al., 2025; Lu & Le, 2025).

Despite demonstrating significant advantages in technical principles and simulation studies, grid-forming microgrids have not achieved expected commercialization and application speeds in practical engineering. Large-scale deployment still faces numerous obstacles. For instance, high initial investment and maintenance costs (Ahmed et al., 2025); complex system control and protection technologies (Lin et al., 2022); unclear or inconsistent grid integration standards and operational rules with existing power grids (Rathnayake et al., 2021); and limited maturity and availability of critical equipment (such as large-capacity grid-forming inverters) (Lin et al., 2022). These challenges do not stem solely from technical bottlenecks. Many deep-seated obstacles extend beyond the technical realm into institutional, market, and societal domains. Specifically: Policy and regulatory frameworks exhibit uncertainty or lag, lacking long-term stable incentives (Ahmed et al., 2025); Immature electricity market mechanisms make it difficult to accurately measure and commercially compensate the diverse ancillary services provided by microgrids (Ali et al., 2017); Coordination challenges across sectors and among multiple stakeholders (such as grid companies, investors, users, and governments) persist, with unclear delineation of rights, responsibilities, and interests (Soshinskaya et al., 2014).

The development challenges of grid-forming microgrids represent a classic “technical–economic–social” complex problem. To overcome its deployment bottlenecks, efforts must extend beyond technological optimization. A systemic, multi-stakeholder perspective is essential to comprehensively analyze the interactions among non-technical factors—including policy, market dynamics, regulations, and social acceptance—to build an ecosystem that collaboratively drives its sustainable development.

### 2.2. Stakeholder Theory in Energy System Transitions

Stakeholder theory posits that outcomes in any organization or complex system are not determined by a single entity but are shaped by the collective goals, behaviors, and interactions of multiple parties with vested interests (i.e., “stakeholders”). This theory provides a relational framework for analyzing systemic change (Donaldson & Preston, 1995; Mitchell et al., 1997). In energy infrastructure projects, core stakeholders typically include governments, businesses, users, and local communities (Ruggiero et al., 2014). Focusing on grid-connected microgrids, key stakeholders primarily include grid operators, investors, end-users, and government agencies (González et al., 2018; Romankiewicz, 2013). Among these, grid operators’ primary concern is system security and stability (Klucznik & Lubośny, 2012); investors pursue long-term economic returns and risk control (Bodewes et al., 2024); consumers expect high-quality, affordable electricity services (M. A. Brown et al., 2020); governments must balance multiple policy objectives including energy transition, economic development, and social equity (Carley & Konisky, 2020).

The transition of energy systems toward low-carbon and decentralized models represents a quintessential socio-technical transformation process. Its successful implementation depends not only on technological feasibility but also on the interaction of market incentives, institutional arrangements, and behavioral responses, thereby constituting a multi-stakeholder coordination problem rather than a purely engineering optimization task (Bekebrede et al., 2018; Sovacool & Geels, 2016). Existing research indicates that collaboration, conflict, and consensus formation among stakeholders are critical determinants of renewable energy project success (Maqbool et al., 2020). Compared to centralized power plants, grid-forming microgrids exhibit stronger territorial embeddedness and technological interactivity (Hicks & Ison, 2018). Their planning, construction, and operation more directly involve local grid characteristics, regional investment patterns, and consumption models, requiring deeper and more frequent localized coordination among heterogeneous actors.

However, a dominant strand of the literature continues to rely on techno-economic optimization logic, implicitly assuming that deployment outcomes follow from linear improvements in technical efficiency, cost reduction, or engineering superiority. This perspective underestimates the fact that, in decentralized energy systems, technical readiness is increasingly no longer the binding constraint. Instead, the primary bottlenecks are shifting toward socio-behavioral and institutional frictions embedded in stakeholder decision-making processes, including investor loss aversion, user acceptance uncertainty, and utility organizational inertia.

Existing research on microgrid stakeholders predominantly focuses on qualitative identification, role description, or theoretical framework development, or concentrates on macro-level analysis of policy and market mechanisms (Carpintero-Rentería et al., 2019; Suryani et al., 2025). Research quantitatively analyzing specific cognitive preferences, decision-making behaviors, and dynamic interactions among different stakeholders remains relatively scarce. However, the operational performance of grid-forming microgrids is critically shaped by the coordinated behaviors of key actors, including utility management practices, investor decision logics, user adoption behavior, and government regulatory design.

Therefore, it is necessary to integrate the systemic perspective of stakeholder theory with micro-level behavioral analysis of key actors in order to move beyond descriptive mapping toward explanatory and predictive modeling of microgrid development outcomes.

### 2.3. Factors Influencing the Development of Grid-Forming Microgrids

In examining factors influencing microgrid development, existing literature establishes an analytical framework across four dimensions: end-users, investors, government agencies, and grid companies. User acceptance primarily focuses on five aspects: physical health and safety, resource utilization in the surrounding area, energy prices, energy supply security and reliability, and environmental awareness (Irfan et al., 2021; Upreti & van der Horst, 2004; Yuan et al., 2015; Zaharuddin et al., 2025; Zoellner et al., 2008). Investor engagement emphasizes technological maturity, technical feasibility, project profitability, user affordability, and market potential (Paravantis et al., 2018; Roopnarain & Adeleke, 2017; Yaqoot et al., 2016). Government support encompasses industrial policy, fiscal incentives, public acceptance, economic benefits, environmental benefits, and employment impacts (Soshinskaya et al., 2014; West et al., 2010). Grid company involvement pertains to the complementary relationship between microgrids and the main grid, project approvals and grid connection permits, revenue mechanisms, and development initiative.

The factors influencing microgrid development do not exist in isolation. Instead, they interact and influence the development process through the dynamics and negotiations among key stakeholders, including users, investors, governments, and grid companies.

## 3. Conceptual Framework and Hypothesis Development

**Figure 1:**
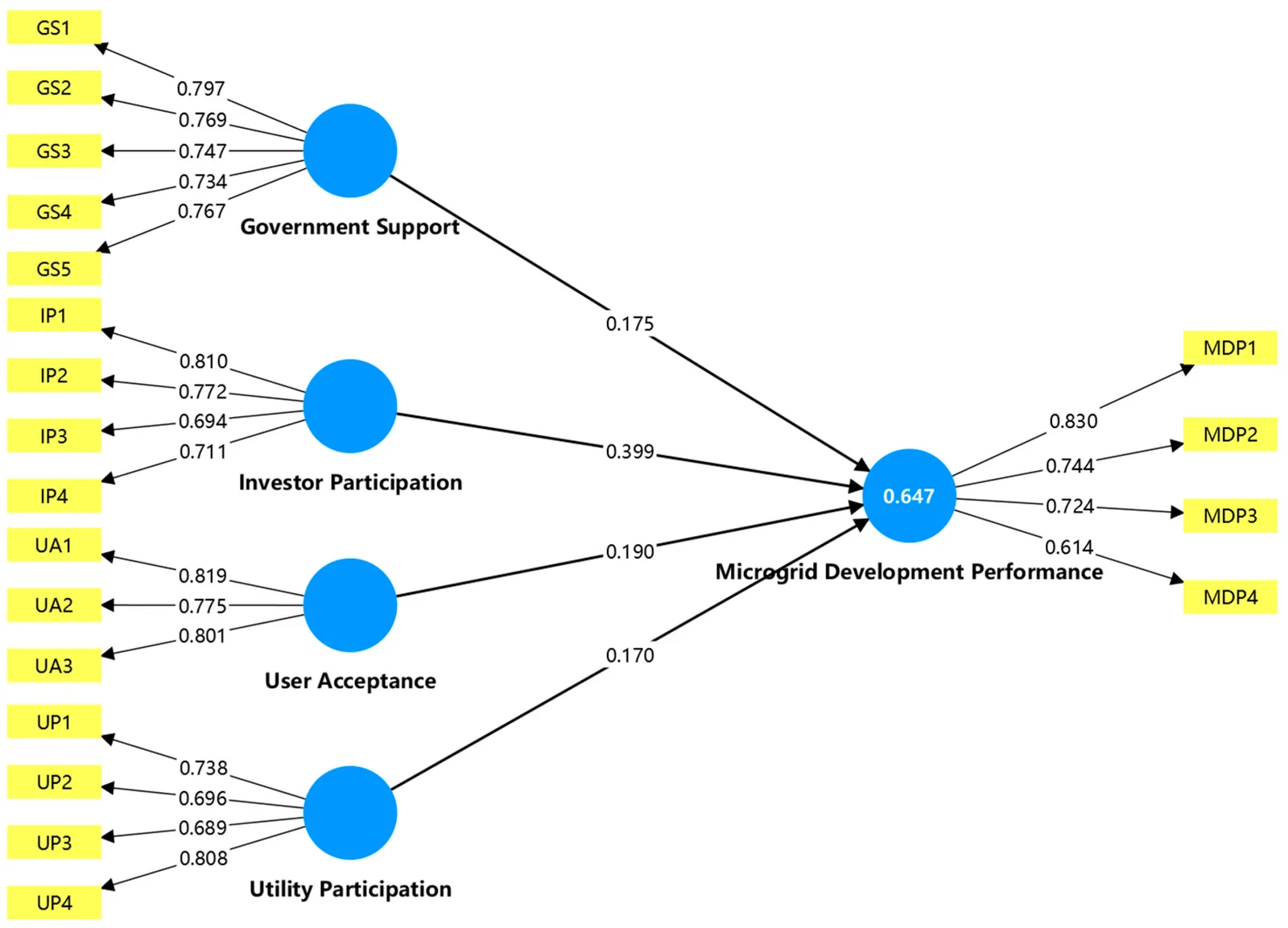
Results of the PLS-SEM.

This study draws on stakeholder theory to develop an analytical framework that examines the behavioral, institutional, and economic determinants of grid-forming microgrid development performance. Stakeholder theory posits that the performance of complex organizational and technological systems is not determined solely by internal technical attributes but by the goals, resources, and coordinated actions of the diverse actors with a stake in system outcomes (Freeman, 2010). This perspective is particularly apt for grid-forming microgrids, which are located at the intersection of technical infrastructure, market dynamics, regulatory governance, and community engagement. Consistent with socio-technical transition theory, which characterizes the adoption of new energy systems as a process jointly shaped by engineering, institutional, and behavioral forces (Wüstenhagen et al., 2007), this study conceptualizes microgrid development performance as a function of four stakeholder-based constructs: government support, investor participation, user acceptance, and utility participation. Figure 1 presents the proposed framework.

### 3.1. Government Support and Microgrid Development Performance

Government actors establish the institutional environment within which grid-forming microgrids are planned, financed, and deployed. Through the formulation of policy instruments, regulatory standards, and financial incentive mechanisms, governments shape the risk landscape that other stakeholders face, reduce transaction costs, and coordinate expectations across actors with divergent interests. Prior empirical research consistently demonstrates that stable and supportive regulatory environments are among the primary enablers of renewable energy investment and deployment (Painuly, 2001; Del Río & Mir-Artigues, 2014). In the context of grid-forming microgrids, this role carries added weight, as these systems frequently operate under hybrid regulatory arrangements that differ materially from those governing conventional centralized infrastructure. When governments provide clear policy signals, coherent legal frameworks, and dedicated support mechanisms, they lower the barriers to stakeholder coordination and resource mobilization that are otherwise prevalent in novel energy system deployment. The absence of such support, conversely, increases project uncertainty and elevates the risk exposure of downstream stakeholders. This reasoning supports the following hypothesis:

**H1.**  *Government support is positively associated with microgrid development performance.*

### 3.2. Investor Participation and Microgrid Development Performance

Capital provision is a necessary precondition for the physical realization of grid-forming microgrids, which typically require substantial upfront investment in generation assets, storage infrastructure, power electronics, and control systems, with payback periods extending across multiple years or decades. The willingness of investors to commit capital to such projects depends on their assessment of financial returns, policy stability, and downside risk, all of which are conditioned by the broader institutional and market environment (Bürer & Wüstenhagen, 2009; Polzin et al., 2019). Investor participation, however, extends beyond the provision of financial capital. Active and sustained investor engagement confers market legitimacy on decentralized energy projects, reinforces perceptions of economic viability among co-stakeholders, and supports the operational continuity necessary for long-term performance. When investor confidence is high, capital mobilization is facilitated, project timelines are more reliably maintained, and the financial capacity required to adapt to operational challenges is more readily available. These mechanisms collectively support improved development outcomes. Accordingly, this study proposes:

**H2.**  *Investor participation is positively associated with microgrid development performance.*

### 3.3. User Acceptance and Microgrid Development Performance

User acceptance constitutes the primary behavioral determinant of grid-forming microgrid performance at the demand side of the system. Decentralized energy configurations fundamentally alter the role of end users, who are no longer passive recipients of electricity supplied by a centralized utility but active participants whose behavioral responses to system operation, energy pricing, and demand-side management directly affect system balance and reliability. Research on the social dimensions of energy transitions establishes that public and user acceptance is a necessary, rather than merely desirable, condition for the sustained performance of renewable and distributed energy systems (Huijts et al., 2012; Sovacool & Ratan, 2012). In the specific context of grid-forming microgrids, user acceptance shapes load profiles, willingness to participate in demand flexibility programs, and tolerance for the operational adjustments required during islanded or transitional operating modes. Users who regard microgrids as reliable, personally beneficial, and aligned with their environmental values are more likely to engage constructively with system management protocols, thereby stabilizing demand-side behavior and supporting overall performance. This reasoning yields the following hypothesis:

**H3.**  *User acceptance is positively associated with microgrid development performance.*

### 3.4. Utility Participation and Microgrid Development Performance

Despite their operational capacity for autonomous functioning, grid-forming microgrids remain embedded within broader energy system architectures that require ongoing coordination with utility operators. Utilities govern the physical and commercial interfaces between distributed systems and the main grid, including interconnection standards, metering arrangements, ancillary service markets, and voltage management protocols. Prior research identifies utility orientation as a critical structural variable in decentralized energy transitions, with utilities capable of either enabling or constraining the integration of distributed resources depending on their strategic posture and regulatory incentives (Markard & Truffer, 2006; Burger & Luke, 2017). When utilities actively engage with microgrid operators, they facilitate smooth grid interconnection, support flexible operational modes, and reduce the technical and administrative friction associated with system integration. In contrast, passive or adversarial utility engagement increases integration costs, delays commissioning, and limits the operational scope available to microgrid developers. Consequently, this study proposes:

**H4.**  *Utility participation is positively associated with microgrid development performance.*

The four hypotheses collectively define a stakeholder-based model in which government support, investor participation, user acceptance, and utility participation each exert a theoretically grounded and empirically testable influence on microgrid development performance. While each construct operates through a distinct mechanism, namely institutional risk reduction, capital mobilization, behavioral demand-side engagement, and operational system integration, they are interrelated in practice: government policy shapes investor risk assessments, investor commitment conditions the resources available for user engagement, and utility cooperation determines the degree to which user and investor expectations can be operationally fulfilled. By examining these four dimensions simultaneously within a single analytical framework, this study provides a more complete account of the stakeholder dynamics governing grid-forming microgrid development than prior research, which has typically addressed these factors in isolation.

### 3.5. A Behavioral Economics Lens on Stakeholder Dynamics

To understand the mechanisms driving microgrid development performance, this study maps the stakeholder constructs onto key behavioral economics principles. Traditional technology adoption models often assume perfectly rational actors; however, decentralized energy transitions are governed by bounded rationality, risk, and cognitive biases. In this context, Government Support functions as an uncertainty-reduction mechanism, providing clear policy heuristics that mitigate the bounded rationality of market actors. Investor Participation is heavily governed by risk perception and loss aversion, as the irreversible nature of capital-intensive microgrid infrastructure makes investors disproportionately sensitive to the downside risk of stranded assets. User Acceptance relies on overcoming status quo bias and establishing trust in the reliability of autonomous, islanded operations. Finally, Utility Participation is frequently constrained by institutional loss aversion and bounded rationality, as legacy operators navigate the perceived threat to centralized control and adapt to new, co-operative integration heuristics. By mapping these behavioral concepts onto our constructs, this framework provides a more robust explanation of the friction and drivers inherent in socio-technical transitions.

## 4. Materials and Methods

### 4.1. Participants

Survey data were collected from a sample of stakeholders with direct involvement in, or substantive familiarity with, grid-forming microgrid development projects. Respondents were drawn from relevant stakeholder populations, including government agency personnel, investment professionals, utility operators, and end users engaged in microgrid-related activities. Following data screening to remove incomplete and inconsistent responses, 200 valid questionnaires were retained for analysis. The demographic and professional characteristics of the final sample are presented in Table 1.

The sample size of 200 meets the minimum threshold established for consistent PLS-SEM estimation. Specifically, it satisfies the ten-times rule of thumb recommended by Hair et al. (2019), whereby the minimum required sample size is determined by multiplying ten by the maximum number of structural paths directed at any single latent construct in the model. This criterion ensures that the sample provides sufficient statistical information for stable path coefficient estimation and reliable hypothesis evaluation.

Participation was voluntary throughout. Before completing the questionnaire, all respondents were informed of the study’s academic purpose, the anonymous nature of data collection, and their right to withdraw at any point without obligation. No personally identifiable information was collected, and all responses were treated as strictly confidential in accordance with standard research ethics protocols.

### 4.2. Procedure and Measures

Data were collected using a structured self-administered questionnaire distributed through an online survey platform. Respondents were recruited from stakeholder populations with professional experience in, or substantive familiarity with, microgrid-related activities, including energy planning, investment, regulation, utility operation, and electricity consumption. To ensure the relevance and quality of the data, a screening question was administered at the outset of the survey instrument. Only respondents who demonstrated a basic understanding of grid-forming microgrids or comparable distributed energy projects were permitted to proceed to the full questionnaire. All survey items were designated as mandatory, thereby eliminating missing data at the item level and ensuring the completeness of each retained response. Respondents were informed of the academic purpose of the study and the voluntary and anonymous nature of their participation before accessing the questionnaire.

The survey instrument was originally developed in English and subsequently translated into Chinese to accommodate the target respondent population. A back-translation procedure was employed to identify and minimize semantic discrepancies between the two language versions (Brislin, 1970). Prior to the main data collection, a pilot test was conducted with a small group of respondents possessing relevant experience in the energy sector. Feedback from the pilot test was used to refine item wording, improve clarity, and confirm the contextual appropriateness of the measurement instrument. Expert review of the instrument was also conducted to assess content validity. Minor linguistic revisions were made to the English items following this review to improve technical precision and interpretability.

All constructs were operationalized using multi-item reflective scales adapted from established literature on renewable energy policy, stakeholder participation, investment behavior, and technology acceptance, with item wording adjusted to reflect the operational and governance context of grid-forming microgrids. The final instrument comprised 20 items spanning five latent constructs: Government Support, Investor Participation, User Acceptance, Utility Participation, and Microgrid Development Performance. Each item was rated on a five-point Likert scale ranging from 1 (“strongly disagree”) to 5 (“strongly agree”).

Government Support (GS; five items: GS1-GS5) was operationalized based on renewable energy policy perception research (Painuly, 2001; Del Río & Burguillo, 2008), capturing stakeholder perceptions of policy stability, financial incentives, and regulatory transparency. Investor Participation (IP; four items: IP1–IP4) was adapted from research on investment behavior in renewable energy contexts (Bürer & Wüstenhagen, 2009; Polzin et al., 2019), reflecting respondents’ assessments of technical confidence, expected market returns, and willingness to assume operational and financial risk. User Acceptance (UA; three items: UA1–UA3) was derived from social acceptability and technology adoption frameworks in the renewable energy literature (Wüstenhagen et al., 2007; Jaaffar et al., 2022), with items capturing perceived safety, economic affordability, and alignment with environmental values. Utility Participation (UP; four items: UP1–UP4) was adapted from distributed energy and utility engagement research (Burger & Luke, 2017; Gomes et al., 2022), measuring the perceived willingness of grid operators to provide operational support, technical coordination, and cooperative integration with grid-forming microgrids. Microgrid Development Performance (MDP; four items: MDP1–MDP4) served as the dependent construct and was adapted from prior research on microgrid performance evaluation and community-oriented energy service delivery (Kaczmarski, 2022). Items assessed functional effectiveness, system resilience, and overall development capacity.

While these latent constructs are rooted in the socio-technical transitions literature, their manifest indicators simultaneously operationalize key behavioral economics principles. For Investor Participation, items assessing the willingness to assume operational and financial risk and technical confidence serve as direct empirical proxies for investor risk perception and loss aversion in capital-intensive settings. For User Acceptance, the measurement of perceived safety functions as a critical indicator of technological trust, while assessments of economic affordability and environmental alignment capture the behavioral utility required to overcome the status quo bias inherent in traditional centralized grid consumption. Consequently, the existing measurement instrument sufficiently captures the micro-level behavioral mechanisms driving the macro-level stakeholder dynamics.

### 4.3. Data Analysis

The proposed research model was evaluated using Structural Equation Modeling (SEM), a multivariate analytical technique that simultaneously estimates measurement and structural relationships among latent constructs. Two principal variants of SEM are employed in the empirical literature: covariance-based SEM (CB-SEM) and Partial Least Squares SEM (PLS-SEM). Although CB-SEM is prevalent in confirmatory research contexts, PLS-SEM has attracted growing scholarly attention for prediction-oriented research designs, its capacity to handle moderate sample sizes, and its comparatively relaxed distributional assumptions (Wen et al., 2023; Hair et al., 2019).

This study employs PLS-SEM over alternative approaches, such as Covariance-Based SEM (CB-SEM), due to its distinct alignment with our research objectives. While CB-SEM is optimal for confirming established, restrictive theoretical covariance structures, the primary analytical objective of this study is explanatory and predictive—aiming to identify the relative structural weights of emerging stakeholder dynamics on performance. Furthermore, PLS-SEM is methodologically superior for models estimating complex structural pathways with a moderate sample size (*N* = 200), as it maximizes explained variance without imposing the strict multivariate normality assumptions required by maximum likelihood estimation in CB-SEM.

The measurement model was evaluated against four criteria: indicator reliability, internal consistency reliability, convergent validity, and discriminant validity. Indicator reliability was assessed by examining the outer loadings of each item on its respective construct. As reported in Table 2, the majority of loadings exceeded the recommended threshold of 0.70, indicating adequate item-level reliability across constructs. Internal consistency reliability was assessed using both Cronbach’s alpha and composite reliability (CR). All constructs returned Cronbach’s alpha values within the acceptable range of 0.70 to 0.95 (Elmustapha et al., 2018), and all CR values exceeded 0.70 (Bagozzi & Yi, 1988), collectively supporting measurement instrument reliability. Convergent validity was evaluated using the average variance extracted (AVE). All constructs yielded AVE values above 0.50, indicating that each latent variable accounted for more variance in its indicators than was attributable to measurement error.

Discriminant validity was assessed to confirm that each construct captured a phenomenon empirically distinct from those represented by the other constructs in the model. Following the Fornell–Larcker criterion, the square root of each construct’s AVE, displayed on the bold diagonal in Table 3, was compared against the inter-construct correlation coefficients in the corresponding row and column. In all cases, the square root of the AVE exceeded the off-diagonal correlations, indicating that each latent variable shared greater variance with its own indicators than with any other construct in the model. For example, the AVE square root for Government Support (0.763) exceeded its correlations with Investor Participation (0.732), User Acceptance (0.614), Utility Participation (0.639), and Microgrid Development Performance (0.693). Comparable patterns were observed for all remaining constructs, confirming adequate discriminant validity throughout the measurement model. Furthermore, given the conceptual proximity and high correlation between Government Support and Investor Participation, strict adherence to the Fornell–Larcker criterion was essential to rule out the risk of overlapping perceptions. Because the AVE square roots for both Government Support and Investor Participation exceed their inter-construct correlation, the data confirms that respondents successfully differentiated the macro-institutional conditions (policy) from meso-level market behaviors (investment risk tolerance), confirming robust empirical distinctness.

To address potential concerns regarding common method bias (CMB), which may arise due to the use of self-reported survey data collected from a single source, this study conducted a full collinearity assessment using variance inflation factors (VIFs) within the PLS-SEM framework. Compared to traditional approaches, the full collinearity VIF approach has been recommended as a more comprehensive and reliable diagnostic for simultaneously assessing both common method bias and multicollinearity in variance-based structural equation modeling (Kock, 2015). Following this approach, all measurement items were included in a full collinearity diagnostic model in which each latent construct was regressed on a common criterion structure. The resulting VIF values were examined against the conservative threshold of 3.3. The results indicate that all VIF values fall well below this threshold, the largest VIF value is 1.872, suggesting that neither pathological collinearity nor common method bias poses a serious threat to the validity of the model estimates in this study. Accordingly, the findings provide empirical support that the observed relationships among constructs are unlikely to be significantly inflated by systematic measurement error associated with the single-source survey design.

Following confirmation of measurement model adequacy, path coefficients representing the hypothesized relationships between the four stakeholder constructs and Microgrid Development Performance were estimated using PLS-SEM. The significance of each path coefficient was evaluated via bootstrapping with 5000 subsamples (Hair et al., 2019). The results, presented in Table 4, support all four proposed hypotheses: Government Support, Investor Participation, User Acceptance, and Utility Participation each exert statistically significant positive effects on Microgrid Development Performance.

Among the four constructs, Investor Participation yielded the largest path coefficient, indicating its dominant role in driving microgrid development performance. This pattern reflects the central importance of capital commitment, investment confidence, and risk-bearing capacity in enabling the deployment and sustained operation of grid-forming microgrids. User Acceptance ranked second in terms of effect magnitude, demonstrating that users’ perceptions of system reliability, economic affordability, and environmental benefits exert a measurable influence on development outcomes. Government Support produced the third-largest effect, indicating that policy stability, regulatory transparency, and institutional incentives remain meaningful enabling conditions, though their direct effect is comparatively smaller than those of investor- and user-side dynamics. Utility Participation, while the smallest in magnitude among the four constructs, returned a statistically significant path coefficient, confirming the relevance of grid operators’ involvement in system coordination, operational support, and grid interaction.

The predictive accuracy of the structural model was evaluated using the coefficient of determination (R^2^) for the endogenous construct. The R^2^ value for Microgrid Development Performance was 0.647, exceeding the conventional benchmark of 0.25 (Hair et al., 2019) and indicating that the four stakeholder constructs collectively accounted for 64.7% of the variance in the dependent variable. Figure 1 presents the full PLS-SEM path diagram, displaying the relationships among latent constructs and the corresponding indicator loadings. The convergence of adequate indicator loadings, acceptable reliability and validity statistics, and statistically significant path coefficients collectively supports the robustness of the estimated model.

## 5. Discussion

### 5.1. Main Findings and Theoretical Implications

The empirical results of this study advance the stakeholder-oriented literature on decentralized energy transitions by establishing the relative behavioral and institutional determinants of grid-forming microgrid development performance. Rather than confirming a purely policy-driven model of infrastructure deployment, the findings reveal a differentiated hierarchy of stakeholder influence in which institutional support establishes enabling conditions, while market and behavioral mechanisms determine operational performance. Several interrelated findings warrant discussion.

While institutional theory traditionally positions government support (H1) as the dominant prerequisite for infrastructure development, the present results indicate a more differentiated functional hierarchy among stakeholders. Government support functions as a critical boundary-setting mechanism that establishes the regulatory and institutional conditions necessary for project initiation, but it does not serve as the primary driver of performance variance once these baseline conditions are in place. Instead, investor participation (beta = 0.399) and user acceptance (beta = 0.190) carry the highest structural weight, suggesting that development outcomes are predominantly determined by capital mobilization and demand-side behavioral engagement. This pattern is consistent with socio-technical transition theory, which conceptualizes policy support as a foundational but enabling condition rather than a direct operational driver of system performance (Geels, 2019; Rogge & Reichardt, 2016). Accordingly, the observed hierarchy reflects a structural shift in decentralized energy systems, where marginal performance variation is increasingly shaped by financial and behavioral mechanisms rather than institutional presence alone.

The dominant role of investor participation indicates that capital confidence, risk tolerance, and long-term return expectations are the most consequential determinants of microgrid development performance in the present sample. This finding extends prior research on private investment in decentralized energy systems (Bürer & Wüstenhagen, 2009; Polzin et al., 2019) by situating investment decisions within the specific context of grid-forming microgrids, where capital intensity and long payback periods significantly elevate the importance of financial commitment. More broadly, the result highlights that development performance is closely tied to financial market conditions and investor behavioral dispositions, underscoring the centrality of capital mobilization in infrastructure realization.

The finding that investor participation exerts a stronger direct effect on development performance than government support warrants explicit clarification, as it may appear counterintuitive in policy-driven energy transition contexts. This pattern reflects the distinction between enabling conditions and execution mechanisms within socio-technical systems. Government policies define the regulatory and institutional boundaries within which projects operate, whereas investors directly finance the physical assets required for system deployment. Because grid-forming microgrids depend on substantial upfront capital investment, the willingness of investors to assume financial risk naturally exerts a more immediate influence on measurable performance outcomes than the presence of supportive policy frameworks. Government support lowers barriers and reduces uncertainty, but it does not physically construct infrastructure or sustain operational cash flows. As a result, the direct statistical impact of investor participation on development performance is expected to exceed that of government support, even though both constructs remain essential components of the overall system. It should also be noted that the designation of government support as H1 reflects its structural position within the conceptual model rather than an expectation of statistical dominance. In socio-technical frameworks, variables are typically introduced sequentially from macro-level institutional conditions to meso-level market actors and micro-level users. The empirical hierarchy observed in the present study therefore represents a functional ordering of performance drivers rather than a contradiction of theoretical assumptions.

User acceptance ranks second in effect magnitude, reinforcing the importance of demand-side engagement in decentralized energy systems. This finding aligns with the social acceptability literature, which emphasizes behavioral commitment and public willingness as necessary conditions for the sustained performance of renewable energy technologies (Wüstenhagen et al., 2007; Sovacool & Ratan, 2012). In the operational context of grid-forming microgrids, users interact directly with energy generation and management systems, making behavioral alignment a direct determinant of system performance. This positions user engagement as an active governance variable within decentralized energy systems rather than a passive outcome of technological deployment.

Government support yields a positive and statistically significant effect but ranks below both investor participation and user acceptance. This result reflects its role as an enabling institutional condition that reduces uncertainty and establishes regulatory stability, rather than a direct source of performance differentiation across projects. In mature stages of deployment, once institutional frameworks become standardized, variation in outcomes is more strongly associated with market behavior and user engagement than with policy presence alone.

Utility participation produces a positive and statistically significant effect that is comparatively modest relative to the other three constructs. This result reflects the structural repositioning of grid operators in hybrid decentralized energy architectures, in which utilities are transitioning from centralized control functions toward cooperative roles centered on system integration, grid interconnection, and market coordination (Burger & Luke, 2017; D. Brown et al., 2020). Grid-forming microgrids possess the technical capability to autonomously regulate voltage and frequency and to operate in islanded mode without continuous reliance on the main grid. This capability fundamentally alters the functional relationship between utilities and end users. Rather than serving as exclusive gatekeepers of electricity delivery, utilities increasingly assume cooperative roles centered on system integration, interconnection coordination, and market facilitation. Their contribution to performance therefore manifests primarily through efficiency and reliability enhancements rather than basic service provision. Consequently, the comparatively smaller path coefficient associated with utility participation does not indicate diminished relevance but rather signals a transition from centralized control to collaborative coordination. This shift reflects the broader evolution of energy systems toward distributed, resilient, and locally managed architectures.

In addition to conceptual and technological explanations, the modest path coefficient for utility participation is partially attributable to the statistical restriction of range. As indicated by the descriptive statistics, utility participation exhibited the lowest variance among all constructs (SD = 0.448). This homogenous response pattern reflects the highly standardized, uniform nature of grid interconnection protocols managed by state-dominated utilities, which inherently attenuates the construct’s explanatory power regarding performance differences across individual projects. While it may initially appear counterintuitive that utility participation yields the lowest path coefficient, this result perfectly encapsulates the paradigm shift introduced by grid-forming technologies. Unlike conventional systems where utilities are the absolute gatekeepers of service delivery, grid-forming microgrids possess the technical capacity to autonomously regulate voltage and frequency, allowing them to provide services to end-users even in islanded modes. Consequently, the utility’s role transitions from a primary service provider to a cooperative system integrator. Their participation remains vital for optimizing grid interconnection and market coordination, but it is no longer a strict prerequisite for localized service provision, thus explaining its comparatively lower structural weight relative to capital and user adoption.

Furthermore, the observed hierarchy of path coefficients must be contextualized within the specific institutional environment of the Chinese energy sector. In China’s state-led framework, top–down policy backing is typically a ubiquitous prerequisite; because it functions as a homogenous macro-constant across most initiatives, it naturally explains less variance in individual project outcomes. Conversely, as Chinese regulators have phased out renewable energy subsidies in favor of a ‘grid-parity’ market model, the financial risk has shifted heavily onto project developers. This structural transition elevates Investor Participation to the dominant determinant of performance. Finally, the comparatively modest effect of Utility Participation reflects the institutional friction inherent in integrating decentralized systems into a grid historically dominated by highly centralized, state-owned monopolies, prompting many microgrid developers to prioritize behind-the-meter autonomy over utility integration.

Collectively, the findings support a reorientation of the analytical lens through which microgrid development is understood, moving beyond purely technical explanations toward a socio-technical framework in which institutional, financial, and behavioral factors jointly determine system performance. Conventional technology adoption models implicitly assume a direct correspondence between engineering capability and deployment outcomes. The present results challenge this assumption by demonstrating that capital confidence, demand-side acceptance, and stakeholder coordination account for a substantial proportion of explained variance in development performance. This contribution broadens the theoretical scope of microgrid research by highlighting the importance of stakeholder alignment in decentralized energy transitions. Rather than treating policy, finance, and user behavior as secondary considerations, the results indicate that these factors constitute core drivers of infrastructure success. Future research may build on this perspective by examining how changes in regulatory regimes, financing mechanisms, and user engagement strategies reshape the dynamics of emerging energy systems.

### 5.2. Limitations and Future Research

This study is not without limitations. First, while the sample adequately represents the aggregate microgrid ecosystem, the distribution of occupational backgrounds may influence the structural weightings.

Second, due to sample size constraints for sub-group partitioning, this study did not execute a formal Multi-Group Analysis (PLS-MGA). Future research should employ stratified sampling to secure sufficiently large sub-samples across distinct stakeholder categories (e.g., utility operators versus private investors) to statistically test for group differences in path estimations.

Third, the present study employs a parallel direct-effects model to establish the baseline relative impact magnitudes of the four stakeholder groups. Consequently, the model inherently assumes linear and independent relationships between the constructs and development performance. In reality, socio-technical transitions are highly interdependent. It is theoretically highly probable that moderating or mediating effects exist—for instance, macro-level government support likely operates as an exogenous antecedent that indirectly influences performance by mediating meso-level investor participation. Because our cross-sectional design is optimized for baseline parallel estimation, future research should employ longitudinal data and hierarchical structural models to formally test these sequential mediations and interaction effects, thereby mapping the causal chain of stakeholder interdependence.

Fourth, the data were collected within the Chinese institutional and regulatory context, which is characterized by state-led energy transition policies and a distinctive utility governance structure. While this setting provides a valuable empirical context for studying grid-forming microgrids at scale, it may limit the generalizability of findings to regions with different regulatory regimes, market maturity levels, or institutional logics. Future studies should replicate the proposed framework in alternative contexts, such as liberalized energy markets in Europe or the United States, to assess external validity.

Fifth, this study relies on self-reported survey data, which reflect stakeholder perceptions rather than purely objective operational metrics. While this approach is consistent with the behavioral economics perspective adopted in this study—particularly in measuring constructs such as risk tolerance, capital confidence, and acceptance behavior—it may be subject to cognitive and perceptual biases. Future research should combine perceptual survey data with objective performance indicators, such as system reliability, frequency stability, or realized financial returns, to enable triangulation of results.

Finally, future research could further extend this work by examining the temporal evolution of stakeholder interactions as microgrid technologies and governance structures mature, as well as by formally modeling the sequential causal pathways linking institutional support, investment behavior, user engagement, and system-level performance outcomes.

## 6. Conclusions and Policy Implications

This study develops and empirically tests a stakeholder-based framework examining the determinants of grid-forming microgrid development performance, drawing on stakeholder theory and integrating behavioral and institutional perspectives through a PLS-SEM approach. The results establish that investor participation is the most consequential driver of microgrid development performance, followed in order by user acceptance, government support, and utility participation. Collectively, these findings demonstrate that the alignment of financial incentives, demand-side behavioral acceptance, and institutional frameworks shapes the viability of technically complex grid-forming microgrids to a degree that extends beyond technological preparedness alone.

Beyond statistical significance, the relative magnitudes of the estimated path coefficients and effect sizes provide important practical insights into the operational hierarchy of stakeholders in microgrid development. The dominant effect of investor participation indicates that capital mobilization represents the decisive enabling condition for project realization. In practical terms, even technologically advanced systems are unlikely to progress without financial arrangements that provide sufficient capital confidence and risk-bearing capacity for high upfront investments. The meaningful effect of user acceptance further demonstrates that users function as active participants whose behavioral responses directly influence system stability and demand alignment. This finding implies that user engagement should be treated as a core design variable rather than a passive consequence of infrastructure deployment. By contrast, the comparatively smaller effect sizes associated with government support and utility participation suggest that these actors operate primarily as enabling or boundary-setting conditions. While necessary to remove institutional and operational barriers, these factors alone are unlikely to generate high development performance in the absence of strong investor confidence and sustained user participation.

This study contributes to the growing literature on socio-technical energy transitions by establishing the distinct and differential roles of key stakeholder groups in determining decentralized energy system outcomes. The results indicate that grid-forming microgrids advance not only in response to policy mandates but also through the accumulation of market confidence and behavioral legitimacy among investors and users. By demonstrating that behavioral and institutional factors account for a preponderance of explained variance in development performance, this study provides empirical support for the socio-technical framing of energy transitions and extends its application to the specific governance context of grid-forming microgrids.

The findings carry four concrete implications for microgrid governance and policy design. First, given that investor participation exerts the largest effect on development performance, policymakers should prioritize the construction of stable and predictable investment environments. Practical mechanisms include long-term revenue certainty instruments (such as fixed-price contracts or feed-in tariff arrangements), risk-sharing structures between public actors and private capital, and transparent regulatory frameworks that reduce uncertainty over project approval timelines and grid access rights. These conditions are particularly consequential in capital-intensive microgrid contexts, where investor confidence must be established well in advance of project execution.

Second, the finding that user acceptance exerts a stronger effect on development performance than government support demands a paradigm shift in how policymakers approach decentralized energy transitions. Historically, energy policy has often conflated institutional support with public adoption. Our results empirically decouple these dimensions, demonstrating that the user’s behavioral perspective operates as an independent and more heavily weighted driver of system success. Consequently, policymakers must seriously consider the user’s perspective as a distinct priority, transitioning from treating users as passive recipients of centralized energy to viewing them as active co-creators of grid stability. To achieve this, governance frameworks must deliberately decouple community engagement from traditional regulatory push. Policymakers should champion participatory design processes that allow end-users to shape local system parameters, ensure transparent communication of direct economic and environmental benefits, and design behavioral incentive structures that reward flexible electricity consumption. If policy frameworks fail to independently cultivate this demand-side acceptance, even robust, top–down government support will be insufficient to guarantee optimal microgrid development.

Third, although government support remains a meaningful enabling condition, its comparatively smaller direct effect relative to investor participation and user acceptance suggests that policy instruments function primarily as boundary-setting mechanisms rather than as direct substitutes for market and behavioral drivers. This implies that policy design should focus on removing structural barriers and creating permissive regulatory environments rather than attempting to substitute mandated deployment for market-driven adoption. As microgrid markets mature, the leverage point for governance intervention shifts progressively from directive policy instruments toward facilitative ones.

Fourth, the positive but comparatively modest effect of utility participation indicates that the repositioning of traditional grid operators from centralized controllers to cooperative system integrators is an ongoing and incomplete process. Policy frameworks should create clear institutional pathways and commercial incentives for utilities to engage constructively with microgrid operators, including standardized interconnection protocols, compensation mechanisms for ancillary service provision, and regulatory recognition of cooperative grid management arrangements within hybrid grid–microgrid architectures (Burger & Luke, 2017; D. Brown et al., 2020).

Taken together, these implications suggest that advancing grid-forming microgrid development requires a transition from state-centric, directive governance models toward stakeholder-coordinated frameworks that cultivate investor confidence, promote user engagement, and support adaptive utility cooperation within a coherent and predictable institutional environment.

## Figures and Tables

**Table 1:** Profile of respondents (*N* = 200).

Variables	Category	Frequency	Percentage
Gender	Male	83	41.5%
	Female	117	58.5%
Age	21–30	69	34.5%
	31–40	106	53%
	41–50	17	8.5%
	>50	8	4%
Educational level	Associate or Below	21	10.5%
	Bachelor	112	56%
	Master	62	31%
	Doctoral	5	2.5%
Occupation	State-Owned Enterprise	39	19.5%
	Privately Owned Enterprise	133	66.5%
	Joint Venture or Wholly Foreign-Owned Enterprise	11	5.5%
	Research Institute or University	11	5.5%
	Other	6	3%
Position	Senior	14	7%
	Middle	84	42%
	First-Line	102	51%
Work tenure	<3	28	14%
	3–5	41	20.5%
	6–7	24	12%
	8–9	39	19.5%
	>9	68	34%
Familiarity with grid-forming microgrid	Unfamiliar	4	2%
	Somewhat Familiar	68	34%
	Familiar	105	52.5%
	Very familiar	23	11.5%

**Table 2:** Model assessment.

Variables	Items	Loading	Cronbach-α	AVE	CR
GS	GS1	0.797	0.821	0.582	0.874
	GS2	0.769			
	GS3	0.747			
	GS4	0.734			
	GS5	0.767			
IP	IP1	0.810	0.736	0.560	0.835
	IP2	0.772			
	IP3	0.694			
	IP4	0.711			
UA	UA1	0.819	0.715	0.638	0.841
	UA2	0.775			
	UA3	0.801			
UP	UP1	0.738	0.713	0.540	0.824
	UP2	0.696			
	UP3	0.689			
	UP4	0.808			
MDP	MDP1	0.830	0.706	0.536	0.820
	MDP2	0.744			
	MDP3	0.724			
	MDP4	0.614			

Note: GS = government support; IP = investor participation; UA = user acceptance; UP = utility participation; MDP = microgrid development performance.

**Table 3:** Descriptive statistics, discriminant validity and correlations.

Variables	Mean	SD	1	2	3	4	5
1. GS	4.15	0.592	0.763				
2. IP	4.26	0.504	0.732 **	0.748			
3. UA	4.28	0.506	0.614 **	0.605 **	0.799		
4. UP	4.28	0.448	0.639 **	0.639 **	0.488 **	0.735	
5. MDP	4.34	0.474	0.693 **	0.731 **	0.622 **	0.630 **	0.732

Note: GS = government support; IP = investor participation; UA = user acceptance; UP = utility participation; MDP = microgrid development performance; ** *p* < 0.05.

**Table 4:** PLS-SEM estimation.

Hypothesis	Hypothesized Path	Path Coefficients	SE	T-Stat.	Prob.	Decision	f^2^
H1	GS→MDP	0.175	0.055	2.444	0.015	Supported	0.033
H2	IP→MDP	0.399	0.064	5.703	<0.001	Supported	0.175
H3	UA→MDP	0.190	0.053	3.453	<0.001	Supported	0.058
H4	UP→MDP	0.170	0.063	3.040	0.003	Supported	0.043
R^2^	0.647	Adj. R^2^	0.639				

Note: GS = government support; IP = investor participation; UA = user acceptance; UP = utility participation; MDP = microgrid development performance.

## Data Availability

The main data and models generated or used during the study appear in the submitted article; the others are available from the corresponding author, on request.

## Abbreviations

The following abbreviations are used in this manuscript:

GS	Government support
IP	Investor participation
UA	User acceptance
UP	Utility participation
MDP	Microgrid development performance
