# Correction: Tang et al. (2026). A Stakeholder-Based Analysis of Factors Influencing the Development of Grid-Forming Microgrids: A Partial Least Squares SEM Approach. *Behavioral Sciences*, *16*(5), 641

**DOI:** 10.3390/bs16101779

**Published:** 2026-09-29

**Authors:** Chao Tang, Jiabo Gou, Xiaoqiao Liao, Jinhua Wu, Hongning Chu, Qingming Wang, Jiaming Fang, Shen Yan

**Affiliations:** 1 State Grid Panzhihua Electric Power Supply Company, Panzhihua 617067, China; tangc121x@sc.sgcc.com.cn (C.T.); goujb6734@sc.sgcc.com.cn (J.G.); liaoxq4857@sc.sgcc.com.cn (X.L.); wujh7216@sc.sgcc.com.cn (J.W.); chuhn0056@sc.sgcc.com.cn (H.C.); wangqm6414@sc.sgcc.com.cn (Q.W.); 2 School of Economics and Management, University of Electronic Science and Technology of China, Chengdu 611731, China; jmfang@uestc.edu.cn

## Missing Funding

In the original publication (Tang et al., 2026), the funder “Science and Technology Project of State Grid Sichuan Electric Power Company (Demonstration Application of an Integrated Grid-Forming Microgrid with Hydropower, Photovoltaic, Energy Storage, DC and Flexible Loads), ERP No. 521913250001” of Chao Tang and Shen Yan was not included.

**Funding:** This work was supported by the Science and Technology Project of State Grid Sichuan Electric Power Company (Demonstration Application of an Integrated Grid-Forming Microgrid with Hydropower, Photovoltaic, Energy Storage, DC and Flexible Loads, ERP No.: 521913250001).

The authors state that the scientific conclusions are unaffected. This correction was approved by the Academic Editor. The original publication has also been updated.
